# Chronic infection during placental malaria is associated with up-regulation of cycloxygenase-2

**DOI:** 10.1186/1475-2875-9-45

**Published:** 2010-02-09

**Authors:** Demba Sarr, Delphine Aldebert, Laurence Marrama, Emilie Frealle, Alioune Gaye, Hamoud O Brahim, Makhtar Niang, Jean Marie Dangou, Odile Mercereau-Puijalon, Jean Yves Lehesran, Ronan Jambou

**Affiliations:** 1 Institut Pasteur de Dakar PoBox 220 Dakar, Senegal; 2 Centre de Santé Roi Baudoin, Guediawaye, Dakar, Senegal; 3 Institut Pasteur, CNRS URA 2581, Paris, France; 4 UR10, Mother & Child Unit, IRD PoBox 1386 Dakar, Senegal; 5 Département de Parasitologie-Mycologie, Institut Pasteur, Institut Pasteur, 28 rue Dr Roux, 75015 Paris, France

## Abstract

**Background:**

Placental malaria (PM) is associated with poor foetal development, but the pathophysiological processes involved are poorly understood. Cyclooxygenase (COX) and lipoxygenase (LOX) which convert fatty acids to prostaglandins and leukotrienes, play important roles in pregnancy and foetal development. COX-2, currently targeted by specific drugs, plays a dual role as it associates with both pre-eclampsia pathology and recovery during infection. The role of COX during PM was questioned by quantifying at delivery COX-1, COX-2, 15-LOX, and IL-10 expression in two groups of malaria infected and uninfected placenta.

**Methods:**

Placental biopsies were collected at delivery for mRNA isolation and quantification, using real time PCR.

**Results:**

COX-2 and IL-10 mRNAs increased mainly during chronic infections (nine- and five-times, respectively), whereas COX-1 transcripts remained constant. COX-2 over-expression was associated with a higher birth weight of the baby, but with a lower rate of haemoglobin of the mother. It was associated with a macrophage infiltration of the placenta and with a low haemozoin infiltration. In the opposite way, placental infection was associated with lower expression of 15-LOX mRNA. A high degree of haemozoin deposition correlates with low birth weight and decreased expression of COX-2.

**Conclusion:**

These data provide evidence that COX-2 and IL-10 are highly induced during chronic infection of the placenta, but were not associated with preterm delivery or low birth weight. The data support the involvement of COX-2 in the recovery phase of the placental infection.

## Background

In Africa, most of the malaria mortality and morbidity is borne by children and pregnant women with an estimate 200,000 infant deaths each year as a result of malaria infection in pregnancy [1]. During placental malaria (PM) parasites accumulate in the placenta with an increased risk of low birth weight (LBW) [2]. This accumulation can be related to low blood flow through the placenta and to specific adhesion of infected red blood cells (IRBCs) on syncytiotrophoblast. This adhesion is mediated by chondroitin sulphate A and specific var antigens. Monocytes also accumulate in the intervillous space of the placenta [3] mostly attracted by release of MIF [4,5]. Pro-inflammatory cytokine release also associates with this accumulation of parasites, pigment, and monocytes in the tissue [6,7].

Decreased blood flow in the placenta is the major factor triggering LBW, which had previously been linked to a modulation of placental cytokine expression [7]. In this context, prostaglandins (PGs) are important modulators of vascular perfusion and fever [8]. Conversion of arachidonic acid to PGs is catalyzed by two isoforms of cyclooxygenase (COX-1 and COX-2) [9]. COX-2-generated PGs are important in inflammation and host defence and is up-regulated prior to the onset of labour [10,11]. Whereas COX-1-generated PGs is implicated in homeostasis and survival of the foetus, and mainly secreted in decidual lining of the uterus [12,13] without modulation during gestation or labour. The role of COX-1 and -2 during placental pathology was suspected some time ago when bacterial lipopolysaccharide mediated foetal death was related to an over-production of COX-2 [14]. In the same line COX-2 over expression was reported during pre-eclampsia [15], but not after this episode [16] and was associated with apoptosis in smooth chorion trophoblast cells of human foetal membrane tissues [17]. This enhancement seems to be triggered by leukocyte microparticles [15,18]. However, Khan *et al *published conflicting results describing a reduction of COX-2 and of the amount of NHE-1 in pre-eclamptic placentas with an unaltered level of COX-1 [19].

During placental malaria, modulation of these PGs pathway could play a role in preterm delivery and low birth weight as observed during pre-eclampsia. A foetal COX-2 gene polymorphism was associated with placental malperfusion during placental malaria [20]. In the general, circulation PGE2 and blood mononuclear cell COX-2 expression were also reported to be inversely related with disease severity in children with malaria [21,22].

Two other arachidonic acid pathways, the epoxide and the lipoxygenase (LOX) pathway [23], are involved in pathology during pregnancy [24]. LOX leads to synthesis of leukotrienes and lipoxins with immune and anti-inflammatory activity. Three enzymes control this pathway: 5-LOX produced by neutrophils, 12-LOX produced by platelets, and 15-LOX located in macrophages and endothelia. 15-LOX is highly inducible and produces hydroxyeicosatetraenoic (HETE) compounds [25]. Interestingly, 15-HETE is a powerful inhibitor of pro-inflammatory eicosanoids, whereas 5-HETE and 12-HETE are chemotactic factors for neutrophils and they also stimulate vasodilation through PG synthesis [26].

Eicosanoids are thus powerful regulators of placenta perfusion and overall of the pregnancy outcome. New analgesic and anti-inflammatory drugs, inhibitors of COX activity, are often used in malaria endemic areas to reduce fever, pain, and inflammation [27]. They can interfere with PGs regulations and modulate the outcome of the pregnancy even if antenatal administration of celecoxib (a COX-2 inhibitor) seems to improve placental perfusion in the pregnant rabbit [28]. All these data pave the way of new studies to understand the pathophysiology of placental malaria, and this study was designed to address the role of these enzymes during PM in low malaria transmission area. The purpose of this study was to evaluate the levels of COX-1, COX-2, and 15-LOX mRNA genes in human placenta infected by *P. falciparum *compared to uninfected placenta. Although delivery is only an instant in the whole pregnancy, it is however a good snapshot of the placenta statement. Studies conducted at delivery also give information on the late stage of the mother-baby interaction.

## Methods

### Placenta collection

Collection of placentas was done at delivery, at the maternity ward the Centre de Santé Roi Baudoin, Guediawaye, Dakar Sénégal. Details regarding enrolment and standard laboratory procedures have been reported elsewhere [29]. It involved women, more than 16 years, with an uncomplicated delivery of live newborns. For all women giving informed consent, an immuno-chromatography test (ICT, PfHRP2 MaKromed) was carried out on placental blood. A standard questionnaire was used to record information including: mother's age, gravidity, gestational age (determined by the date of the last menstrual period and morphometric measurement of the uterus during antenatal clinic visits) and history of malaria attacks. Babies and placentas were weighed immediately after birth. Gender and Apgar scores were recorded. LBW was defined as < 2,500 g, and prematurity as gestational age < 37 weeks. Women with haemoglobin (Hb) less than 11 g/dl were considered anaemic.

The HIV infection status of the participants was not established at the time of the study however, HIV sero-prevalence in Senegalese population was low (0.8% of parturients in Dakar) [30]. All ICT-positive women received chloroquine after delivery. Anaemic women received iron and folic acid supplements.

After collection, whole placentas were refrigerated until assessment (less than two hours after delivery). For each placenta, four biopsies were sampled [31], rinsed in phosphate buffer saline (PBS), rapidly frozen in liquid nitrogen and stored at -80°C until RNA extraction. An additional four biopsies were sampled for histology and immunohistochemistry studies.

### Histology and immunohistochemistry

Immediately after collection, samples for histology and immunohistochemistry were fixed in 4% buffered paraformaldehyde and processed as previously described [29]. 5 μm cryosections were stained either with haematoxylin-eosin (H&E) or with May-Grünewald-Giemsa (MGG). Quantification of parasites was done in placenta for 100 fields (magnification 1000×) of MGG stained sections and in blood by examination of Giemsa-stained thick smears. Haemozoin (HZ) was identified on sections as brown granular material with high refringence on polarization microscope, which differentiates then from formalin crystals. Contain in HZ was qualitatively assessed on sections as: no HZ, low HZ (1-2 crystals/field at ×1000) or high HZ (> 2/field).

Acute infections (AI) were defined by the presence of parasites on sections without malaria pigment, chronic infections (CI) by presence of parasites and pigment, and past infections (PI) by the presence of pigment alone. PfHRP2+ placentas with no evidence of parasites or pigment were classified as "no-parasite" (NP). For the control group all PfHRP2 rapid diagnosis test, parasites and pigment detection were negative in placenta and in the venous blood.

For immunochemistry, after blocking endogenous peroxidases and non-specific binding, tissue sections were incubated for 1 h at room temperature with primary antibodies or control isotypes and revealed using LSAB detection kit (DakoCytomation). Antibodies used were CD68 (PG-M1), and HLA-DR (TAL.1B5) from DakoCytomation mouse antibodies against. The number of cells in the intervillous space was counted over 100 fields (magnitude ×400).

### mRNA quantification

Frozen biopsies were ground into powder in liquid nitrogen before homogenization in 4 M guanidium buffer. RNAs were extracted using standard phenol-chloroform procedure, precipitated with isopropanol and washed before quantification. Integrity of total RNA was assessed on an ethidium bromide (EtBr) stained denaturing agarose gel. For reverse transcription 2 μg of RNA was denatured at 65°C for 10 min, cooled on ice-water, and reverse-transcribed for 60 min at 43°C with 40 U/μl RNase inhibitor and oligo-dT. Amplification was performed in an ABI Prism 7000 Sequence Detection System using Taqman Universal PCR Master Mix (PE Biosystems), containing 300 nM of primers and 200 nM of fluorogenic probes. Each measurement was carried out in duplicate and amplification of Peptidyl Propyl Isomerase A (PPIA, also called Cyclophylin A) was conducted on the same plate. Others housekeeping genes such as beta-2-microglobulin and beta-actin were run together with PPIA, but were not selected for standardization as less constant between infected and uninfected placentas. Probes and primers (Table 1) were designed using Primer Express software (Applied Biosystems). Cycling conditions started at 50°C for 2 minutes followed by 95°C for 10 minutes, then 40 cycles of 95°C for 15 seconds and 60°C for 1 minute. The specificity of PCR amplifications was confirmed for each set of primers by sequencing of the PCR product and primers specificity was confirmed by single peaks demonstrated by dissociation curves after amplification of cDNA. The amplification efficiency was assessed for all the genes by amplifying serial dilutions of cDNA, and calculation of the slope (delta Ct)/(log cDNA dilution) which must be less than 0.1. Quantification of gene expression was done according to Livak *et al *[32] using for each gene the mean Ct-values of the duplicate. For each sample the results were adjusted according to the level of PPIA in the same sample. The PfHRP2-negative control group was used to normalize the results. For each gene, the relative quantity (RQ) of mRNA was expressed, in arbitrary units, as a fold increase in comparison with the control group.

**Table 1:** Nucleotides sequences of the primers and probes used for real time PCR. PPIA: Peptidyl Propyl Isomerase A.

Human Gene	Accession number	Sequences (a: Probe, b: Primer F, c: primer R)	Amplicon size (bp)
COX-1	NM_000962	a) Fam-CTG GCC TCA GCA CTC TGG AAT GAC AA-Tamra b) ATG ATG GGC CTG CTG TGG A c) CCA ACA CTC ACC ATG CCA AAC	150
COX-2	NM_000963	a) Fam-TGC CCA GCA CTT CAC GCA TCA GTT-Tamra b) GCT CAA ACA TGA TGT TTG CAT TC c) GCT GGC CCT CGC TTA TGA	79
15-LOX	NM_001140	a) Fam-TCC ACC AGG CTT CTC TCC AGA TGT CC-Tamra b) GGA GAC AGT GAT GGC GAC ACT c) TCT GCC CAG CTG CCA AGT	74
PPIA	NM_021130	a)Fam-AGC TCA AAG GAG ACG CGG CCC A-Tamra b) GTC AAC CCC ACC GTG TTC TT c) CTG CTG TCT TTG GGA CCT TGT	73
IL-10	NM_000572	a)Fam-CAG GCA ACC TGC CTA ACA TGC TTC-Tamra b) TGA GAA CAG CTG CAC CCA CTT c) ATC TCC GAG ATG CCT TCA GC	70

### Statistical analysis

Differences between groups were compared using Mann-Whitney U test (MW) for two groups or with Kruskal-Wallis test (KW) and Median test for more than two. Correlations were calculated using a Spearman R test (SP). Alpha risk was set at 0.05.

## Results

### Chronic infection is associated with lower birth weight

During this study, 692 pregnant women were examined among whom 71 had a *Plasmodium falciparum *histidine rich protein positive-RDT (PfHRP2+) (10.3%). For these PfHRP2+ women, 25% of the babies had LBW and 67% of the women were anaemic [29]. Compared to the control women, chronic-active infections were associated with lower birth weight (p < 0.008 and p < 0.0002 respectively) as well as CI and PI correlated with lower haemoglobin levels (p < 0.05 for both). No significant change was found for birth weight and haemoglobin, according to parity may be due to the low sample size of the study. For the purpose of this study, 54 placental samples were randomly selected from the different histological groups after classification (table 2). Among the selected PfHRP2+ women, 13 were primiparous, 13 secundiparous, and 14 multiparous.

**Table 2:** Clinical and laboratory data of enrolled women according to the class of infection.

Variables	Ctrl (PfHRP2-)	Total (PfHRP2+)	NP	Past I	Acute I	Chronic I
No. of women	14	40	10	10	10	10
Primigravidae n (%)	4 (28)	13 (32.5)	3 (30)	3 (30)	3 (30)	4 (40)
Secundigravidae n (%)	5 (36)	13 (32.5)	3 (30)	3 (30)	3 (30)	3 (30)
Multigravidae n (%)	5 (36)	14 (35)	4 (40)	4 (40)	4 (40)	3 (30)
Age (years)	24.6 ± 1.6	25.6 ± 1.13	25.2 ± 2.3	26.6 ± 0.6	26.0 ± 1.96	24.8 ± 2.3
Pregnancy duration, weeks	40.8 ± 1.2	40.5 ± 2.6	41.1 ± 1.1	40.9 ± 0.9	41.1 ± 1.1	41.3 ± 1.3
Birth weight (g)	3,153 ± 93	2,723 ± 93*	2,995 ± 137	2,623 ± 131*	2,862 ± 167	2,411 ± 251*
Haemoglobin (g/dl)	11.9 ± 0.3	9.4 ± 2.5*	10.2 ± 1.2	8.5 ± 0.4*	8.4 ± 1.1*	10.2 ± 0.9
Parasitaemia, Venous	0	83%	0	0	205.5%	35.3%
Parasitaemia, Placental	0	294%	0	0	644.5%	%

(*) means significant when compared with the control group (p < 0.05)

NP (no parasites): PfHRP2 positive placenta without parasites or pigment detected by microscopy; PI (past infection): only pigment detected by microscopy; AI (acute infection): only parasites detected; CI (chronic infection): parasites and pigment detected; control (Ctrl): uninfected women with PfHRP2 negative placenta. Data are mean ± SE. Parasitaemia are expressed as parasites per 100 leukocytes. (*) significant difference with control group (p < 0.01 Mann Whitney U test).

### Past and chronic infections are associated with a higher level of COX-2 and IL-10 mRNA

**Figure 1:**
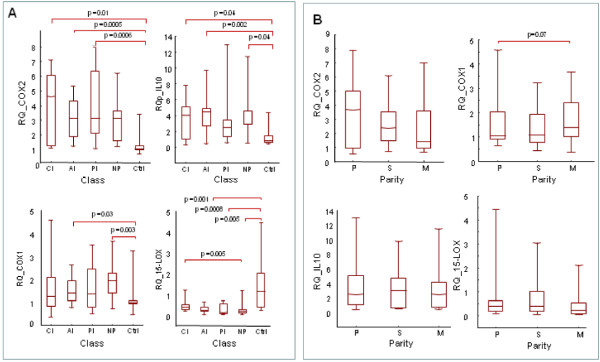
**Expression of COX-1, COX-2, 15-LOX and IL-10 mRNA in placenta**. Expression levels was calibrated for each gene using PPIA as housekeeping gene (delta Ct = Ctgene - CtPPIA) and normalized using the pool of uninfected control placentas (delta delta Ct = delta Ct - mean delta Ct (ctrl)). Results are plotted according to: (A) infection classes: CI = Chronic Infections, AI = Acute Infections, PI = Past Infections, NP = No parasites/pigment detectable, Ctrl = uninfected placentas (PfHRP2-) (B) Parity: P = primiparous, M = multiparous, and S = secondiparous women. RQ = Relative Quantity = 2^-(deltadelta Ct)^. P value are indicated for significant test only (p < 0.05).

COX-2 mRNA increased during PM especially during late stage of the infection: 9.0, 5.4, 3.8 and 3-time higher in placentas with CI, AI, PI and NP, respectively, than in the control group (p < 0.01 MW for CI and AI). The expression of COX-1 remained low in all the placentas without significant differences between PfHRP2- (Ctrl) and PfHRP2+ (CI, AI, PI & NP) groups, but trends to increase during past infection (Figure 1A).

Because IL-10 has been shown to exert direct regulatory effects on prostaglandin production, we also measured IL-10 mRNA in our placental samples. The levels of IL-10 transcripts were higher in PfHRP2+-placentas than in controls (MW p = 0.004, Figure 1A) and correlated with COX-2 expression (n = 54, SP p = 0.006 R = 0.36; and p = 0.008 R = 0.36). However none of these markers correlated with the parasitaemia. In the opposite way 15-LOX was lower in PfHRP2+-placentas than in controls (MW p = 0.0002) (Figure 1A), but correlates with parasitaemia in the placenta (SP R = 0.416, p = 0.0076) and with COX-2 mRNA expression (SP p = 0.018, R = 0.37).

The levels of COX-2 mRNA but not of COX-1, 15-LOX, and IL-10, trend to decrease when parity increased (Figure 1B), but there was no significant difference for any of these genes according to duration of the pregnancy or with the presence of fever at delivery.

### Elevated level of COX-2 is associated with anaemia in the mother but not with low birth weight

**Figure 2:**
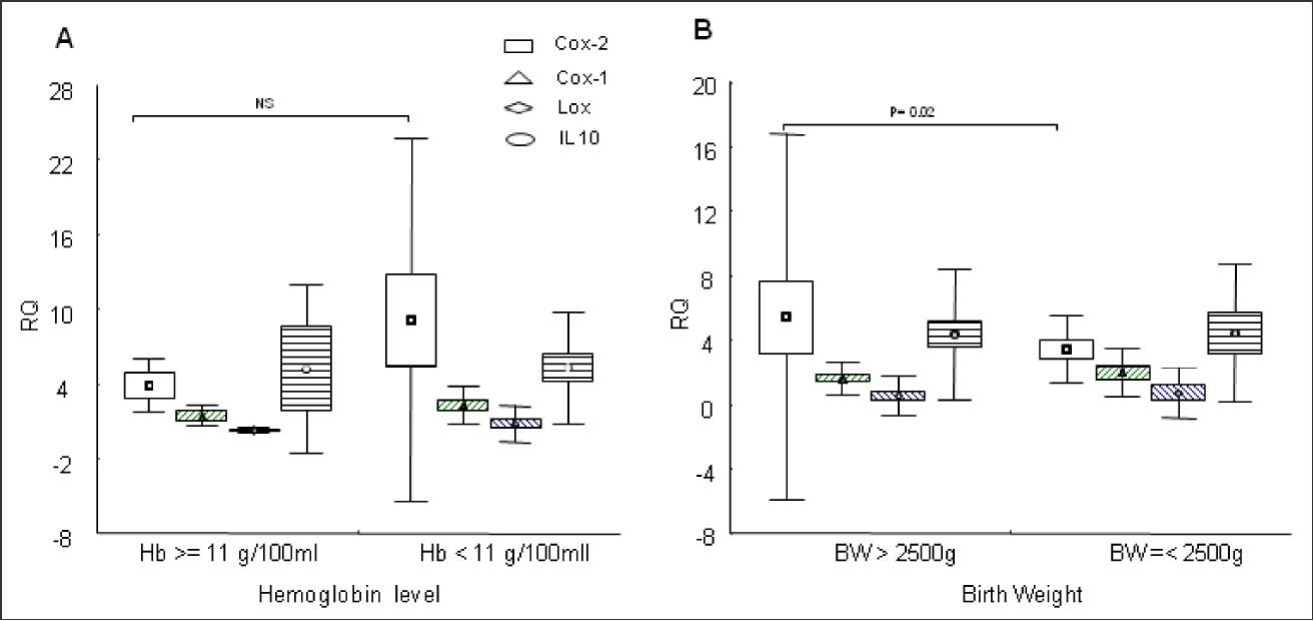
**Expression of COX-1, COX-2, 15-LOX and IL-10 mRNAs in placenta**. Expression levels of each gene (calibrated as in Fig.1) plotted according to (A) haemoglobin level of the mother (g/100 ml) (B) Birth weight of the newborn. Hb = hemoglobin; BW = birth weight; RQ = Relative quantity. P value are indicated for significant test only.

Birth weight was significantly lower in primiparous than in secundiparous or multiparous (MW p < 0.05 for both). It was also lower during AI and CI than in control group. However, women who delivered babies with normal birth weight had higher level of COX-2 transcripts compared to others (MW p < 0.02; Figure 2B). In the opposite way COX-1 and 15-LOX transcripts trend to increase in association with LBW (respectively 2 and 1 fold increase than for the normal birth weight group, NS Figure 2B).

**Figure 3:**
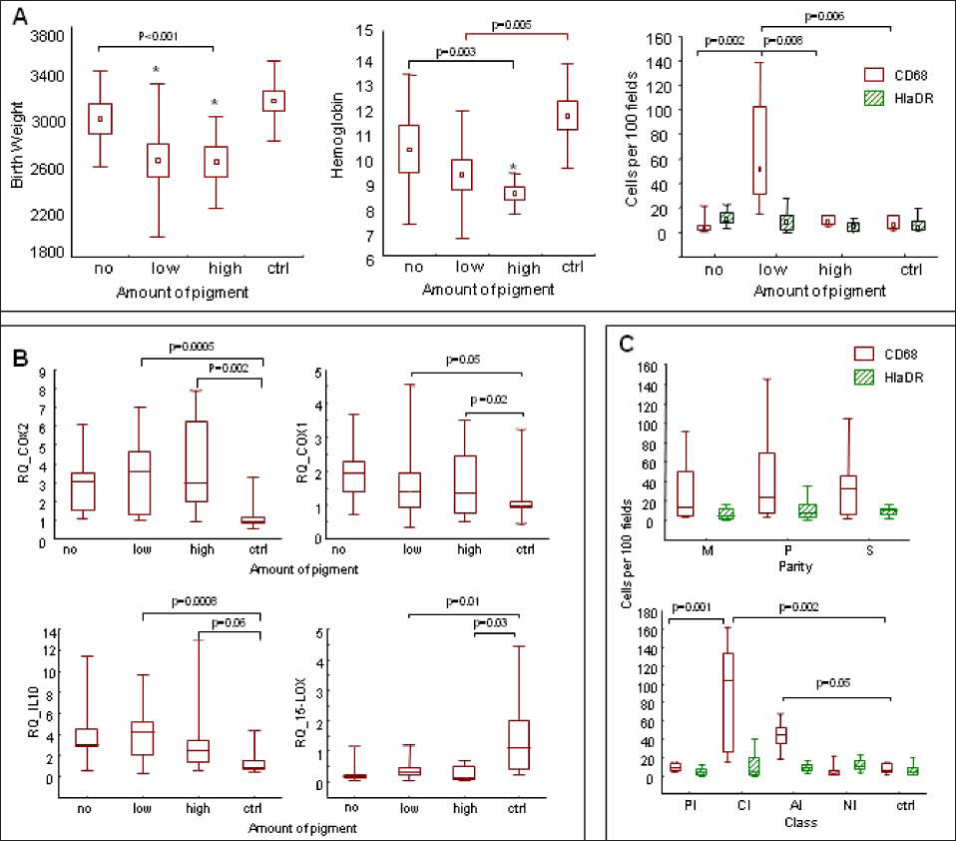
**Impact of the charge of pigment in the placenta**. For PfHRP2+ placenta, the pigment deposit was plotted as: "no pigment" (No); "low load of pigment" (low) for less than 3 pigments for 10 field (×400); "high load of pigment" (high) for more than 3 pigments per field; "control group" (Ctrl) for PfHRP2- placenta. A) Relation between charge in pigment and birth weight of the newborn (g), B) Relation between charge in pigment and haemoglobin level of the mother (g/100 ml), C) Relation between charge in pigment and count of CD68+ and HLA-DR cells in the placenta (number of cells/100 fields at magnification ×400). D) Relation between charge in pigment and expression level of the four genes (RQ = Relative Quantity = 2-(deltadelta Ct)). NS = not significant, (*) means significant when compared with the control group.

Haemoglobin was inversely related to the level of COX-1, COX-2 and IL-10 but increased with 15-LOX (SP R = -0.58, -0.57, -0.51, 0.48 respectively, p < 0.001 for each) (Figure 2A). Haemoglobin tends to decrease with the amount of HZ in the placenta (Figure 3A, middle panel; NS). Anaemia was thus associated with an increase of COX-2 and with PI (MW p < 0.001, with control group).

### COX-2 expression increase with macrophage recruitment and with decrease of pigment deposit

High level of pigment in the placenta was associated with significantly lower birth weight (KW p = 0.013, n = 44, Figure 3A, left panel), and with lower haemoglobin level in the mother (KW p = 0.018, Figure 3A, middle panel). However lower pigment density associated with a higher level of COX-2 and IL-10 transcripts (Figure 3d), without significant change for COX-1 (MW, p < 0.001 for both).

As detected by immunohistochemistry (Figure 3c), macrophages (CD68+) and activated HLADR+ presenting cells increased in PfHRP2+-placenta (p < 0.001 for both, DNS), mainly during late infection (CI or PI versus Ctrl: MW p < 0.001). Primiparous and secondiparous women exhibited higher levels of CD68+ cells when compared to multiparous (p < 0.05) while CI had significantly higher levels of CD68+ cells compared to PfHRP2- or control (p = 0.002). The density of macrophages (CD68) increased in the placenta when the amount of pigment was low (median test p < 0.0001). The count of macrophage was significantly correlated with COX-2 (SP p = 0.014, R = 0.331), IL-10 (SP p = 0.014, R = 0.332) and 15-LOX mRNA (SP p = 0.0003, R = 0.539). In the placental tissue sections COX-1 protein was detected by immunolabelling in trophoblasts, mononuclear interstitial cells and, most often, in foetal endothelial cells. COX-2 and 15-LOX were mainly detected in macrophages, endothelial cells and trophoblasts. During past and chronic infection, COX-2 was highly expressed in trophoblasts (Sarr, personal communication).

## Discussion

This study is part of a project conducted on the pathophysiology of PM in West African urban areas. Despite the low transmission rate of malaria, a high prevalence of PM was observed in this area [29], but its pathogenesis is still poorly understood.

As previously described [11] COX-1 expression remained low in all the samples of our study, in comparison with COX-2. COX-2 expression clearly increased after long-term malaria active infection (CI) of the placenta. Consistent with previous reports [3], chronic infection was associated with a macrophage infiltration of the placenta, which correlated with COX-2 over-expression. In placenta COX-2 was mainly detected in macrophages, vascular lymphocytes and fibroblasts [16], which can explain this correlation. These data indicate that low amount of pigment was associated with the highest levels of COX-2 and IL-10 transcripts, whereas, high amount of HZ was associated with a lower COX-2 expression. In the same line, women with high amount of HZ had babies with lower birth weights and lower level of haemoglobin. This is in line with data showing inhibition of intervillous blood mononuclear cells (IVBMCs) charged in HZ and in particular suppression of PGE2, TNF, and IL-10 secretion [33,34]. This phagocytosis of haemozoin was reported i) to enhance matrix metalloproteinase-9 activity and TNF production in human monocytes [35,36], and ii) to reduce immune response by an activation of a Toll-like receptor 9 mediates mechanism [37]. Overall a low charge in HZ could be a signal to attract macrophages and stimulates cells whereas a high charge could inhibit them.

All these data are consistent with an inflammatory process [3,38]. However, COX-2 (unrelated to the term of the pregnancy) was positively correlated with the birth weight, which supports a protective role of this process for the foetus development. Macrophages can also participate in this protective role, as well as prostacyclins secreted by chorion endothelium, which are known to induce relaxing of foetal vasculature [26]. High level of COX-2 was also previously associated with recovery from anaemia [39]. Along the same lines, IL-10 mRNA was significantly higher in infected placentas compared to uninfected ones, as previously described [40,41]. High level of IL-10 could be deleterious, as IL-10 is known to inhibit COX-2 expression in preterm human placenta [42] and secretion of inflammatory cytokines by monocytes [43]. It was also associated with high parasitaemia, severe anaemia and a low level of COX-2 in PBMC [8,22].

However this could not be relevant during PM as i) COX-2 seems mainly expressed by placenta and not by PBMC and as ii) the effect of IL10 on COX-2 and PGE2 seems to be different in preterm and in term placentas [42]. In the same line, IFN-gamma and oestrogen receptor-beta modulates COX-2 expression in human placental villous endothelial cells [44,45] and need to be measured.

Interestingly, 15-LOX expression correlated with the presence of parasites in the intervillous space, which is consistent with study stating that 15-HETE and 15-HPETE stimulated red blood cell adhesion to endothelium [46]. However 15-LOX mRNA expression remained lower in infected samples compared to uninfected or control samples, which can exclude its role in the sequestration of parasites in the placenta.

## Conclusion

In summary, this study showed that COX-2 and IL-10 are highly expressed in active PM. COX-2 correlates with macrophage infiltration and is related to a better outcome of the pregnancy with higher birth weight. All these data support the increase of COX-2 as a witness of placental defence against infection and draw attention on uncontrolled use of anti-COX2 during placental malaria.

## Abbreviations

PM: placental malaria; COX: cyclooxygenase; LOX: lipooxygenase; LBW: low birth weight; ICT: immunochromatographic test; RDT: rapid diagnosis test; HZ: hemozoin; PBMC: peripheral blood mononuclear cell; CI: Chronic infection; AI: acute infection; PI: past infection; NP: No parasite and pigment (PfHRP2+); PfHRP: *Plasmodium falciparum *histidine rich protein.

## Competing interests

The authors declare that they have no competing interests.

## Authors' contributions

RJ conceived the study. DS and EF performed the experiments. DS, LM, JMD and RJ analysed the data. DS and RJ wrote the manuscript. DA and LM helped revise it. All authors read and approved the final manuscript.
